# Weighted gene co-expression network analysis of the peripheral blood from Amyotrophic Lateral Sclerosis patients

**DOI:** 10.1186/1471-2164-10-405

**Published:** 2009-08-27

**Authors:** Christiaan GJ Saris, Steve Horvath, Paul WJ van Vught, Michael A van Es, Hylke M Blauw, Tova F Fuller, Peter Langfelder, Joseph DeYoung, John HJ Wokke, Jan H Veldink, Leonard H van den Berg, Roel A Ophoff

**Affiliations:** 1 Department of Neurology, Rudolf Magnus Institute of Neuroscience, University Medical Center Utrecht, Utrecht 3584 CX, the Netherlands; 2 Department of Human Genetics, School of Medicine, University of California Los Angeles, Los Angeles, CA 90095, USA; 3 Department of Biostatistics, School of Medicine, University of California Los Angeles, Los Angeles, CA 90095, USA; 4 Semel Institute of Neuroscience and Human Behavioral, School of Medicine, University of California Los Angeles, Los Angeles, CA 90095, USA; 5 Department of Medical Genetics and Rudolf Magnus Institute of Neuroscience, University Medical Center Utrecht, Utrecht 3584 CG, the Netherlands

## Abstract

**Background:**

Amyotrophic Lateral Sclerosis (ALS) is a lethal disorder characterized by progressive degeneration of motor neurons in the brain and spinal cord. Diagnosis is mainly based on clinical symptoms, and there is currently no therapy to stop the disease or slow its progression. Since access to spinal cord tissue is not possible at disease onset, we investigated changes in gene expression profiles in whole blood of ALS patients.

**Results:**

Our transcriptional study showed dramatic changes in blood of ALS patients; 2,300 probes (9.4%) showed significant differential expression in a discovery dataset consisting of 30 ALS patients and 30 healthy controls. Weighted gene co-expression network analysis (WGCNA) was used to find disease-related networks (modules) and disease related hub genes. Two large co-expression modules were found to be associated with ALS. Our findings were replicated in a second (30 patients and 30 controls) and third dataset (63 patients and 63 controls), thereby demonstrating a highly significant and consistent association of two large co-expression modules with ALS disease status. Ingenuity Pathway Analysis of the ALS related module genes implicates enrichment of functional categories related to genetic disorders, neurodegeneration of the nervous system and inflammatory disease. The ALS related modules contain a number of candidate genes possibly involved in pathogenesis of ALS.

**Conclusion:**

This first large-scale blood gene expression study in ALS observed distinct patterns between cases and controls which may provide opportunities for biomarker development as well as new insights into the molecular mechanisms of the disease.

## Background

Amyotrophic lateral sclerosis (ALS) is a devastating disease characterized by progressive degeneration of motor neurons in brain and spinal cord leading to muscle weakness. ALS can occur at anytime in adulthood. Initial manifestations are weakness of limbs, or weakness in the bulbar region leading to abnormalities of speech, swallowing difficulties and facial weakness. The patient eventually becomes paralyzed and approximately 50% of patients die within 3 years after onset of symptoms, usually as the result of respiratory failure. The predominant presentation of ALS is sporadic accounting for >90% of cases whereas familiar ALS affects less than 10% of the patients, usually with autosomal dominant inheritance. Genetic linkage studies have successfully identified several ALS-related genes in the familial forms of the disease. Up to 20% of the familial cases are linked to mutations in the cupper-zinc (Cu/Zn) superoxide dismutase-1 (SOD1) gene on chromosome 21q22.1. Sporadic and familial ALS are clinically indistinguishable except for a slightly earlier age of onset in familial variants.

The pathogenesis of sporadic ALS is largely unknown, but there is emerging evidence that several distinct molecular mechanisms may play a role including oxidative stress, glutamate excitotoxicity, protein misfolding, apoptosis, inflammation, dysfunction of axonal transport, and mitochondrial dysfunction [1]. Recently, four genome-wide genetic association (GWA) studies have been performed to identify common genetic variation involved in susceptibility to sporadic ALS [2-6]. The GWA studies resulted in three new candidate genes for ALS, namely *FGGY *(FGGY carbohydrate kinase domain containing), *ITPR2 *(inositol 1,4,5-triphosphate receptor, type 2) and *DPP6 *(component of type A neuronal transmembrane potassium channels). Recently, a validation study for DPP6 was reported [7]. Other initial findings await further genetic validation, especially given the fact that these studies were performed in relatively small sample sizes compared to GWA studies of other, more prevalent, complex traits [8-10].

While there is evidence of genetic heterogeneity underlying disease susceptibility, clinical manifestations of the devastating ALS phenotype are relatively homogeneous. The latter suggests that at the cellular and molecular level only a limited number of pathways may be involved in disease susceptibility and progression. The identification of molecular pathways related to ALS remains an important challenge, nevertheless. Gene expression studies of spinal cord tissue from human SOD1 transgenic ALS mice and autopsied ALS patients have identified upregulation of genes involved in specific pathogenic pathways such as antioxidant systems, apoptosis and neuroinflammatory cascades [7,11-23]

Drawbacks of previous studies include limited sample size, the use of monogenetic animal models, or human tissue from autopsy at the very end stage of motor neuron degeneration.

As many essential genes and signaling cascades are expressed in blood cells, suggesting that parts of their regulatory networks also exist in blood, we hypothesized that blood gene expression profiles could help elucidate pathways underlying disease etiology. A number of studies describe the search for blood markers for diseases without known clinical phenotypes present in peripheral blood. A proof-of-principle study of blood gene expression profiling in neurological disease was initially performed in a rat model in which a number of neurological conditions (ischemic stroke, hemorrhagic stroke, kainite-induced seizures, hypoxia, or insulin-induced hypoglycaemia) were induced [24]. Different patterns of gene expression were observed in peripheral blood cells one day after each experimental cerebral condition indicating the potential for applying genomic microarray technology to identifying peripheral markers of neurological disease. More recent studies show further evidence that peripheral blood gene expression can be used as a fingerprint of neurological diseases, including Huntington's disease, Alzheimer's disease and autism [25-27].

## Results

### Standard gene expression analysis based on peripheral blood

We collected blood data from three independent patient sets (Table 1). Controls were matched for age and gender, with mean age between 62 and 65 at time of blood collection. We obtained a discovery dataset of 30 patients and 30 controls (dataset 1), a replication dataset of same size (dataset 2) and a third dataset with 63 cases and 63 controls (dataset 3) amounting to a total of 123 ALS patients and 123 controls. Datasets 1 and 2 have similar proportions male/females and spinal/bulbar onset in patients. Dataset 3 has more male subjects (60%) and more spinal onset patients (78%).

**Table 1:** Clinical information on ALS patients and controls.

**Clinical variables**	**Patients**	**Controls**
Number		
Dataset 1	30	30
Dataset 2	30	30
Dataset 3	63	63
Male gender (%)		
Dataset 1	15 (50)	15 (50)
Dataset 2	14 (47)	15 (50)
Dataset 3	38 (60)	40 (63)
Age at blood collection ^¶^		
Dataset 1	63.8 (41.0–76.0)	62.8 (42.8–80.8)
Dataset 2	63.7 (35.3–79.5)	64.8 (36.2–75.8)
Dataset 3	65.0 (23.5–80.8)	64.1 (36.9–81.2)
Age at disease onset ^¶^		
Dataset 1	62.8 (40.5–75.6)	
Dataset 2	62.5 (34.2–78.4)	
Dataset 3	64.4 (21.5–79.6)	
Bulbar Onset (%)		
Dataset 1	15 (50)	
Dataset 2	15 (50)	
Dataset 3	14 (22)	

For the three independent datasets, we report sample sizes and patient characteristics. The close similarity between patients and controls reflects the fact that healthy controls were chosen such that they matched the age and gender of the patients.

¶ Median years (range)

Using a Student t-test for comparing gene expression between ALS cases and healthy controls, we calculated a statistical significance level (p-value) for each of the 24365 probes present in the discovery set. At a false discovery rate of 0.05, 2300 probes are differentially expressed between ALS cases and controls. In Additional File 1, we report the fold change, the p-value, and the Benjamini Hochberg correction for each of these differentially expressed probes. A drawback of a standard analysis is that it ignores the strong correlation patterns between probes, which may lead to an erroneous estimate of the false discovery rate. But a more serious drawback of the standard analysis is that it fails to see the forest for the trees. Below, we will show that two large clusters of genes (modules) relate to ALS disease status. These modules turn out to be highly enriched with known gene ontologies which provides insights into the pathogenesis of ALS.

To predict ALS status based on the gene expression profiles, we used two alternative prediction methods: a random forest predictor and a k-nearest neighbor (with k = 10). Unbiased test set estimates of the prediction accuracy show that both predictors classify 80% of the samples correctly. While the accuracy is relatively high (and reflects the fact that ALS cases are molecularly distinct from controls), it is not clear whether a molecularly predictor of ALS (versus healthy controls) would be clinically relevant. A more basic research question is to identify disease related pathways and gene networks since this may lead to insights regarding the disease etiology and possible treatment regiments. Gene co-expression network methods have been successfully applied in a variety of different settings [28-41].

### Weighted gene co-expression network analysis

Here we used weighted gene co-expression network analysis (WGCNA) [42-45] in a first attempt to identify ALS associated coexpression modules and their key constituents. WGCNA starts from the level of thousands of genes, identifies modules of co-expressed genes, and relates these modules to clinical variables and gene ontology information. Because gene modules may correspond to biological pathways, focusing the analysis on modules (and their highly connected intramodular hub genes) amounts to a biologically meaningful data reduction scheme.

Highly correlated module genes are represented and summarized by their first principal component (which is referred to as the module eigengene [46]). The module eigengenes are used to define measures of module membership which quantify how close a gene is to a given module. Module membership measures allow one to annotate all genes on the array and to screen for disease related intramodular hub genes [44,47,48]. As described below, we use functional enrichment analysis with regard to known gene ontologies to understand the biological significance of module genes and to identify putative disease pathways.

### Detection of co-expression modules related to ALS

**Figure 1:**
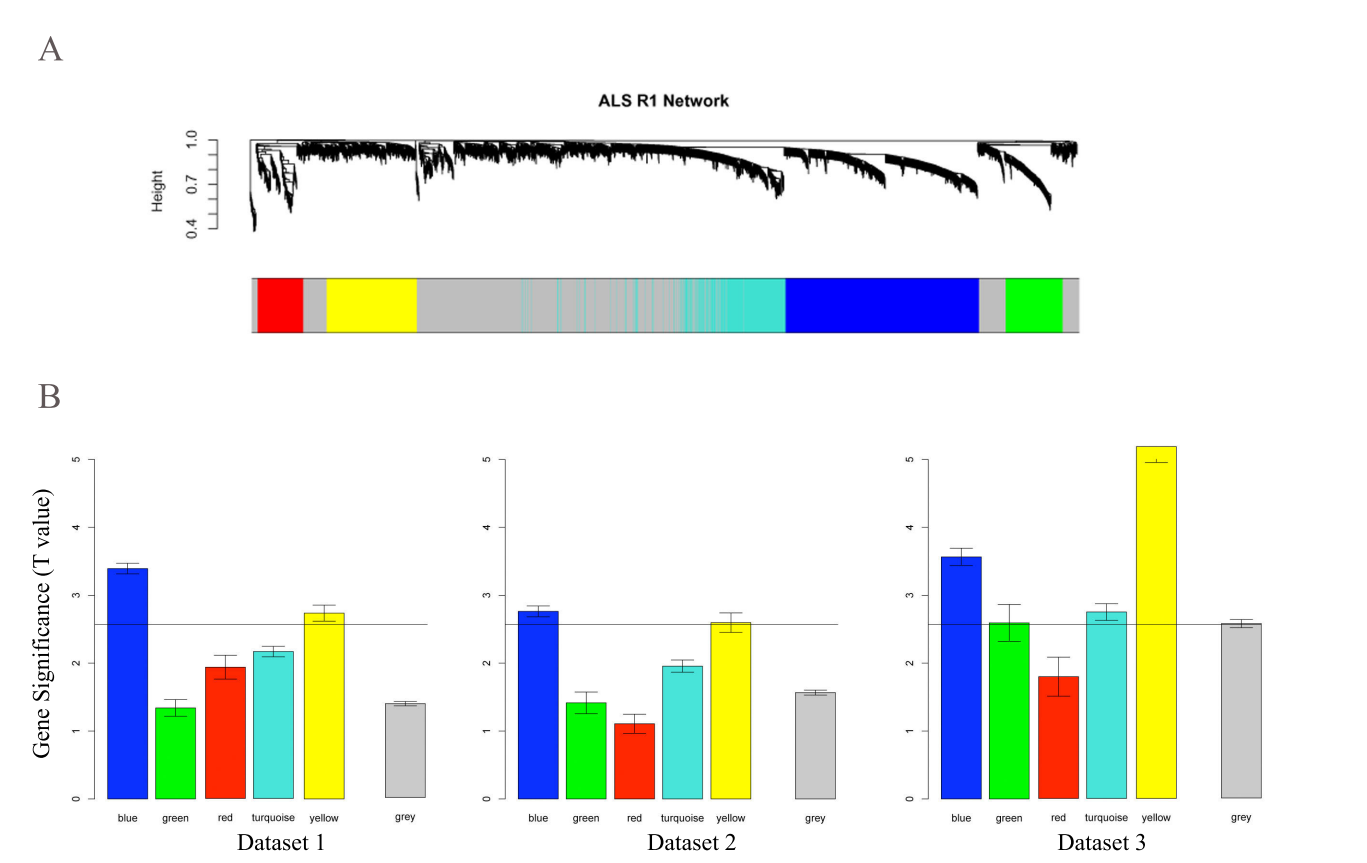
**Gene co-expression modules in human whole blood**. Detection of gene co-expression modules in human whole blood datasets comprised of ALS patients and matched controls. (*a*) Branches of the cluster dendrogram of the most connected genes gave rise to five gene coexpression modules (Blue, Green, Red, Turquoise and Yellow). Genes that could not be clustered into one of these modules were assigned to the Grey module. Every gene represents a line in the hierarchical cluster. Distance between two genes is shown as height on the y-axis. (*b*) Boxplots showing gene significance per module. Module significance was calculated by taking the average of the absolute t statistics of all genes within a module. The Blue and Yellow module were the only modules that were significant at a Bonferroni corrected significance threshold of 0.05/5 in all 3 datasets.

We applied WGCNA to probes with a significant mean detection level (p < 0.05). Hierarchical clustering applied to the discovery set (data set 1) led to the identification of five co-expression modules ranging in size from 199 to 842 genes (Figure 1A). As can be seen from the color-band underneath the cluster tree, modules correspond to branches and are color-coded (Blue, Yellow, Turquoise, Red and Green module). Grey is used to color background genes that are not grouped into any module.

To assess the robustness of the co-expression module definition, we replicated module detection in the second and third dataset (Additional File 2) where we colored the genes according to the module color in data set 1. The fact that genes of the same color stay close together in the three different cluster trees (Additional File 2) shows that the Blue and Yellow module are highly preserved across the three data sets.

### Differentially expressed genes tend to be in the Blue or Yellow module

**Figure 2:**
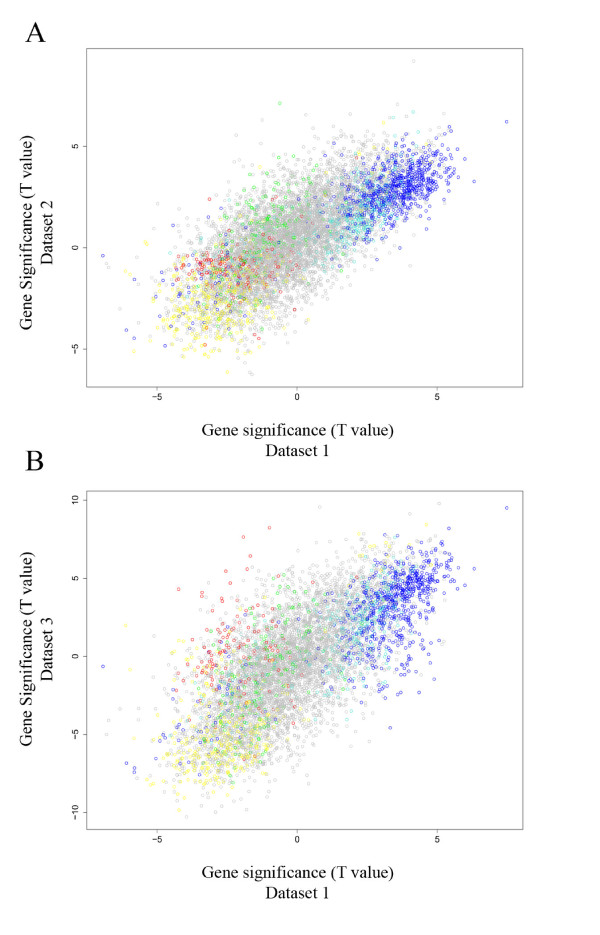
**Scatterplots showing strong preservation of gene significance across the three independent datasets**. The scatterplots include the network genes colored by their module assignment in dataset 1. t-test statistic value for dataset 1 (x-axis) was compared to the t statistic value for dataset 2 (y-axis A) and dataset 3 (y-axis B). Genes that are highly differentially expressed in dataset 1 also tend to be differentially expressed in datasets 2 and 3. Note that Blue genes tend to be over-expressed in ALS patients while yellow genes tend to be under-expressed.

Our module definition was solely based on the gene expression levels in peripheral blood and ignored ALS disease status. To incorporate a clinical outcome into the network analysis, WGCNA makes use of suitably defined gene significance measure. Here we defined the gene significance measure as the Student t-test statistic for testing differential expression between cases and controls. Thus, a large absolute value of the gene significance measure corresponds to a small 2-sided p-value. We found that the gene significance measures in the three independent data were highly correlated (Figure 2A–B: r = 0.73 for dataset 1 vs. dataset 2 with p < 2.2 × 10^-16 ^and r = 0.71 for dataset 1 vs. dataset 3 with p < 2.2 × 10^-16^). Thus, the gene significance measures is highly reproducible across the three data sets. Figure 2A–B shows that genes that are consistently up-regulated in ALS cases tend to be part of the Blue module while genes that are consistently down-regulated tend to be in the Yellow module.

### Two co-expression modules are significantly associated with ALS

We defined a measure of module significance as average absolute gene significance across the module genes. Figure 1B shows that the Blue and Yellow module genes were highly enriched with differentially expressed genes in the 3 independent data sets. The module significance (mean absolute Student t-test statistic) of the Blue module corresponds to p-values 0.0006, 0.005 and 0.0007 in datasets 1, 2, and 3, respectively. The module significance of the Yellow module genes corresponds to p values 0.006, 0.01 and 3.5 × 10^-7 ^in datasets 1, 2 and 3, respectively. Combining all datasets resulted in highly significant p values: 3.0 × 10^-6 ^for the Blue module and 1.3 × 10^-8 ^for the Yellow module.

An alternative and statistically preferable way of relating a module to ALS disease status is to correlate disease status with a suitably defined module representative. As module representative, we used the module eigengene (ME) which is defined as the first principal component of the module expression profiles. The correlations between ALS status and the module eigengene of the Blue module (referred to as MEblue) were r = 0.48 (p = 3.6 × 10^-5^), r = 0.37 (p = 2.6 × 10^-3^), and r = 0.41 (p = 6.2 × 10^-7^) in data sets 1, 2, and 3, respectively. Note that the p-values remain highly significant even after using the most stringent multiple comparison correction (Bonferroni correction) since only 5 comparisons (corresponding to 5 modules) were carried out. This illustrates a statistical advantage of WGCNA: instead of correcting the analysis for tens of thousands of gene comparisons, a module-based analysis involves orders of magnitudes fewer comparisons. For the Yellow module eigengene MEyellow we found highly significant negative correlations with ALS status: r = -0.61 (p = 6.2 × 10^-9^), -0.50 (p = 1.3 × 10^-3^), -0.61 (p < 1.0 × 10^-22^) in data sets 1, 2, and 3, respectively. The negative correlations reflect that most Yellow module genes were under-expressed in ALS patients.

### No significant relationship between modules and other clinical variables

We related the module eigengenes to other clinical variables but did not find any other significant associations. In particular, MEblue and MEyellow were not significantly associated with age at time of collection, gender, specific characteristics of ALS patients such as bulbar or spinal onset, age at onset or El Escorial criteria at time of collection. A multivariate Cox regression analysis that regressed survival time on the module eigengenes, site of onset, sex and age at onset, resulted in no significant p-values for any of the covariates.

### Using module membership values to annotate the genes with regard to module membership in the data sets

As detailed in the Methods section, we made use of a fuzzy measure of module membership (*MM*) that can be defined for each module. The module membership measure with regard to the Blue module *MMblue(i) = Cor(x*_*i*_, *MEblue*) is defined as the correlation between the i-th gene expression profile *x*_*i *_and the Blue module eigengene. Large absolute values of *MMBlue(i) *indicate that the gene is close to (or part of) the Blue module. In contrast, if *MMBlue(i) *is 0, then the i^th ^gene is uncorrelated with the Blue module eigengene and is unlikely to be part of the Blue module. The sign of module membership encodes whether the gene has a positive or a negative relationship with regard to the Blue module expression profiles.

We also used a correlation test to compute a corresponding p-value of module membership. We found that the module membership measures of the Blue and Yellow modules are highly preserved across the three data sets as can be seen from Additional File 3.

In Additional File 4, we report the individual module membership values with regard to the different modules in each of the data sets and the mean module membership values across the three independent data sets is referred to as *MeanMMblue*.

The WGCNA R package also computes a gene selection score (referred to as p.weighted) based on gene significance and module membership [45]. Analogous to a p-value, the smaller the value of p.weighted the stronger is the evidence that the gene is a disease related hub gene.

### Ingenuity pathway analysis of four top 500 gene lists

As detailed in the Methods section, we used the module membership values to define four different gene lists. The first and second gene lists consisted of the top 500 genes closest to the Yellow and the Blue module, respectively. The third gene list consisted of 500 genes with the lowest WGCNA gene selection score p.weighted. For comparison with a standard differential network analysis, we also drafted a fourth list of genes according to the average Student t of differential expression across the three data sets.

In the Methods section and Additional File 4, we provide details on these gene lists including p-values and local false discovery rates (q-values). We used Ingenuity Pathways Analysis (IPA, ) to test for enrichment with regard to known gene ontologies. A detailed comparative functional enrichment study of the four lists is presented in Additional File 5 and a condensed version involving only the first 3 lists can be found in Additional File 6.

We find that a standard differential analysis (black horizontal bars in Additional File 5) leads to less significant findings than those of a module based analysis (see categories cellular compromise, infectious disease, genetic disorder, skeletal/muscular disorder, dermatological diseases, connective tissue disorder). This provides indirect evidence that a module centric analysis of these data leads to more significant biological findings.

### Detailed enrichment analysis results for the Blue module

Here we provide a detailed description of the most important functional enrichment of the 500 genes with highest module membership value in the blue module.

**Post-Translational Modification **was the most significant category with p-values ranging between 4.4 × 10^-4 ^and 4.5 × 10^-2^. Specifically, the following genes are involved in this category: BTG1, BMI1, CAND1, CD47, CD48, CHUK, CLK1, CLK4, CUL2, DNAJA2, DPM1, DUSP11, DUSP12, ELF1, HDAC2, HSP90AA1, MAP3K7, NAE1, PCMT1, PCNP, PPP1CB, PPP1CC, PPP2CA, PPP3CB, PTPN11, RB1CC1, SET, SH2D1A, SIAH1, SIRT1, SLC35A1, SUZ12, UBA3, UBE2N, ZDHHC17. This category included several subcategories, including modification of protein (p = 4.4 × 10^-4^), neddylation of protein (5.9 × 10^-4^), refolding of protein (8.3 × 10^-3^), tyrosine dephosphorylation of protein (8.3 × 10^-3^), moeity attachment of protein (1.1 × 10^-2^), deacetylation of protein (1.5 × 10^-2^), acetylation of protein (4.5 × 10^-2^) and methylation of amino acids (4.5 × 10^-2^).

**Infection Mechanism **was also a highly significant category with p-value range: 5.9 × 10^-4 ^– 4.8 × 10^-2^. In particular, the genes ATG5, BNIP2, CD46, CHUK, DEK, NGLY1, PRNP, RAB11A, SFRS1, TBK1, TFRC, UBP1, WASL (includes EG:8976), WIPF1 and XPO1 were involved in Infection Mechanism. This category included mobility of vaccinia virus (5.9 × 10^-4^), replication of virus (1.9 × 10^-2^), binding of virus (4.6 × 10^-2^), infection of Influenza virus (2.4 × 10^-2^), penetration of human herpesvirus 6A (2.4 × 10^-2^) and penetration of human herpesvirus 6B (2.4 × 10^-2^).

RNA Post-Transcriptional Modification (p-value range 9.5 × 10^-4^-2.8 × 10^-2^) included the following genes: DCP2, DHX15, DNAJB11, DUSP11, EIF4A2, HNRNPH3, IVNS1ABP, NCBP2, PRNP, RNGTT, SFRS1, SFRS2, SFRS3, SFRS6 and SFRS7. This category had subcategories including modification of RNA (9.5 × 10^-4^), splicing of RNA (1.7 × 10^-3^), processing of RNA (3.4 × 10^-3^), decapping of mRNA (2.4 × 10^-2^), dimerization of tRNA-Lys (2.4 × 10^-2^) and selection of splice site (2.8 × 10^-2^)

**Neurological Disorder **(8.5 × 10^-3^-4.8 × 10^-2^) involved the following genes: ACADM, AP1S2, ATP6AP2, B2M, CAB39, CDKN1B, CHMP2B, CRBN, DDX1, EIF3E, GALC, GHITM, GNA13, HMGB2, HMGCR, HSP90AA1, IFNGR1, IMPA1, ITPR1, IVNS1ABP, L1CAM, NDUFB5, NFE2L2, OSBPL8, PCMT1, PPP1CB, PPP1R2, PRNP, PTGS2, RAB1A, RAB11A, RAB3GAP2, RAB5A, SLC9A6 TOMM20 and TRAM1. This category contained subcategories Huntington's disease (1.6 × 10^-2^), atrophy of dendrites (2.4 × 10^-2^), pseudobulbar paralysis (2.4 × 10^-2^) and degeneration of myelin figure (4.8 × 10^-2^).

### Detailed enrichment analysis results for the Yellow module

Here we provide a detailed description of the most important functional enrichment of the 500 genes with highest module membership value in the yellow module.

**Genetic Disorder **was one of the most significant categories with p-values ranging between 8.3 × 10^-7 ^and 4.7 × 10^-2^. Specifically, the following genes are involved in this category: ACADVL, ADA, ALG1, ANAPC2, ATXN2, CD4, CDK9, CDK2AP2, COG1, DPAGT1, FLNA, GSS, GUSB, IDUA, IKBKB, IL2RG, ITGAL, MAP2K5, MAP4K1, MCM5, NOD1, NDUFS7, NDUFS8, NDUFV1, SDHA, SIGIR, SLC35C1, SMPD1 and USP11. This category included several subcategories, including psoriatic arthritis (p = 8.3 × 10^-7^), congenital disorders of glycosylation (2.0 × 10^-4^), Leigh syndrome (2.2 × 10^-4^), and spinocerebellar ataxia, type 2 (2.7 × 10^-2^).

**Neurological Disease **was another significant category with p-value range: 2.0 × 10^-5 ^– 2.7 × 10^-2^. In particular, the following genes CLCN7, CLN3, DIAPH1, HSD17B10, HSP90AB1, HD, NDUFS7, NDUFS8, NDUFV1, NFKB2, SDHA and VAC14 were involved in Neurological Disease. This category included neurodegeneration of nervous system (2.0 × 10^-5^), neurodegeneration of central nervous system (2.7 × 10^-2^), neurodegeneration of peripheral nervous system (2.7 × 10^-2^), Leigh syndrome (2.2 × 10^-4^), Huntington's disease of nervous syndrome (2.7 × 10^-2^), oxidative stress response (2.7 × 10^-2^), fragmentation of striatal neurons (2.7 × 10^-2^) and spinocerebellar ataxia, type 2 (2.7 × 10^-2^).

**Inflammatory Disease **(p-value range 8.3 × 10^-7^-4.0 × 10^-2^) included the following genes: ADA, ANAPC2, CDK9, CDK2AP2, IKBKB, ITGAL, MAP2K5, MAP4K1, MCM5, NOD1, POLD1, SIGIRR and USP11. This category had subcategories including psoriatic arthritis (8.3 × 10^-7^), acute pancreatitis (2.7 × 10^-2^), and keratitis (4.0 × 10^-2^).

**Skeletal and Muscular Disorder **(8.3 × 10^-7^-2.70 × 10-2) involved the following genes: ANAPC2, BIN1, CDK9, CDK2AP2, FLNA, IKBKB, ITGAL, MAP2K5, MAP4K1, MCM5, NOD1, SIGIRR and USI. This category contained subcategories psoriatic arthritis (8.3 × 10^-7^), Melnick-Needles syndrome (2.7 × 10^-2^), disorganization of myofibrils (2.70 × 10^-2^), otopalatodigital syndrome (2.7 × 10^-2^).

### Detailed Ingenuity analysis of the top 500 genes selected by WGCNA

The third list of 500 genes with lowest WGCNA score p.weighted are comprised of genes that are highly related to ALS status and are highly connected intramodular hub genes in the Blue and/or the Yellow module. An Ingenuity analysis of these top 500 genes revealed the following most significant categories: Cellular Compromise (8.5 × 10^-5 ^– 2.8 × 10^-2^) and Post-Translational Modification (9.8 × 10^-5 ^– 5.0 × 10^-2^). **Cellular Compromise **included genes ABCA1, ARHGDIA, CD46, CD55, EXOC7, HMGB2, HD, NFE2L2, PKN1, PLEC1, TBPL1 and VPS26A. This category included degeneration of epithelial cells (8.45 × 10^-5^) and damage of neuromusclar junctions (2.8 × 10^-2^).

**Post-Transcriptional Modification **included 57 genes, and the following subcategories: modification of protein (9.8 × 10^-5^), modification of amino acids (4.7 × 10^-4^), modification of L-proline (4.5 × 10^-3^), modification of L-amino acid (4.8 × 10^-3^), moeity attachment of amino acids (9.7 × 10^-4^), moeity attachment of L-amino acid (6.40 × 10-3), hydroxylation of L-amino acid (4.5 × 10^-3^).

We also applied to the biomarker search option of Ingenuity to these top 500 genes.

The results can be found in Additional File 7 which reports i) tissues where matching genes have been found to be expressed, and ii) known drugs for matching genes. We report these preliminary results to illustrate how WGCNA coupled with Ingenuity Analysis can be used for generating hypotheses that may form the starting point of future studies.

### Functional enrichment analysis with DAVID of the top 100 disease related intramodular hub genes

We also carried out a functional enrichment analysis with the data base DAVID [49]. Here we selected the top 100 most highly connected genes in both modules. Additional File 8 reports highly significant gene ontology categories and representative genes.

The most significant pathway is the Huntington's Disease pathway (Fisher's exact test p = 8.0 × 10^-5^). Other interesting significant pathways include mRNA processing, the neurodegenerative disorders pathway (p = 0.024), the axon guidance pathway (p = 0.025), and the phosphatidylinositol signaling system (p = 0.038).

**Figure 3:**
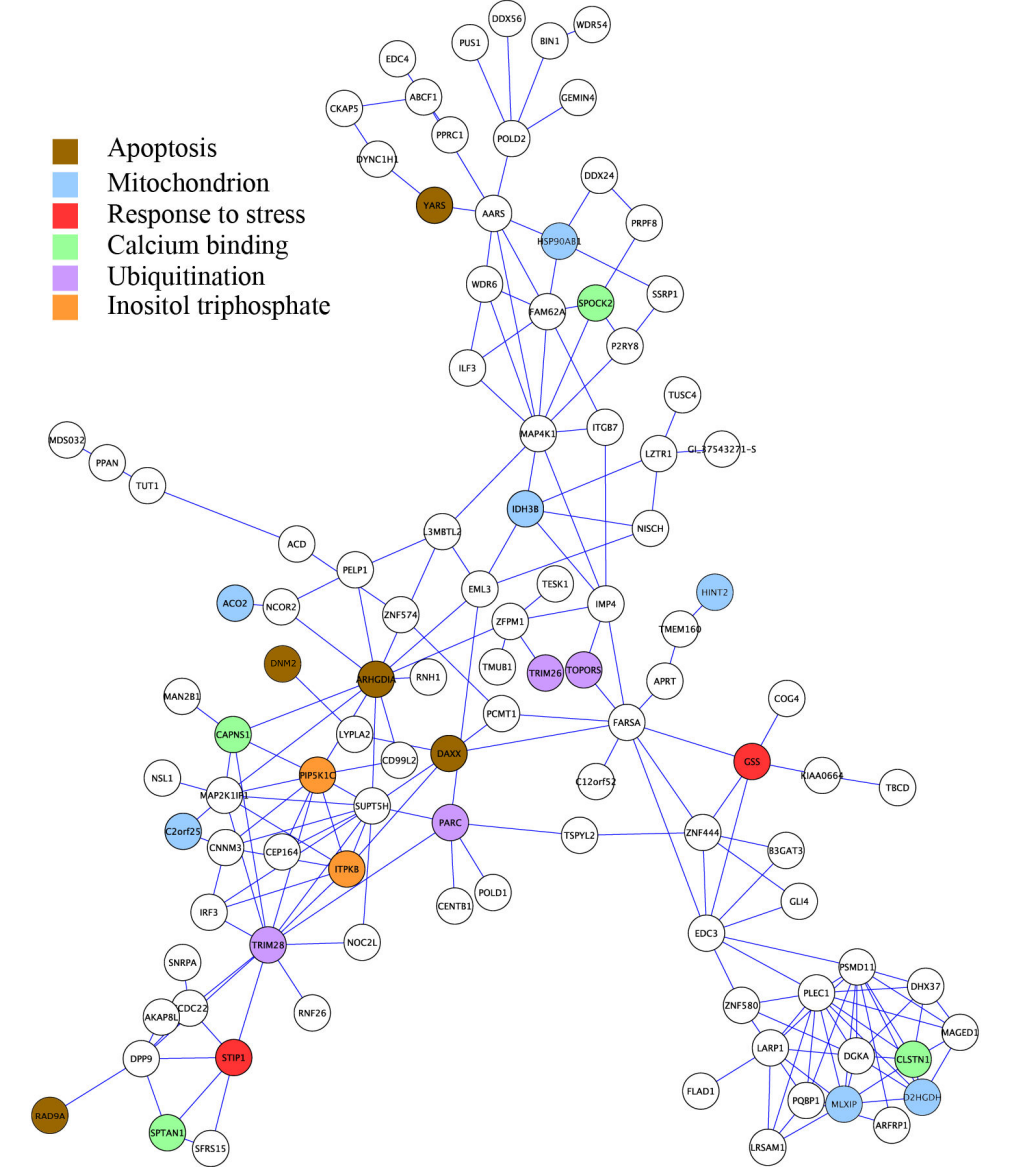
**Network of hub genes in the Yellow module colored by gene ontology functional information**. Hub genes are connected by an edge if the correlation between their expression profiles is significant.

**Figure 4:**
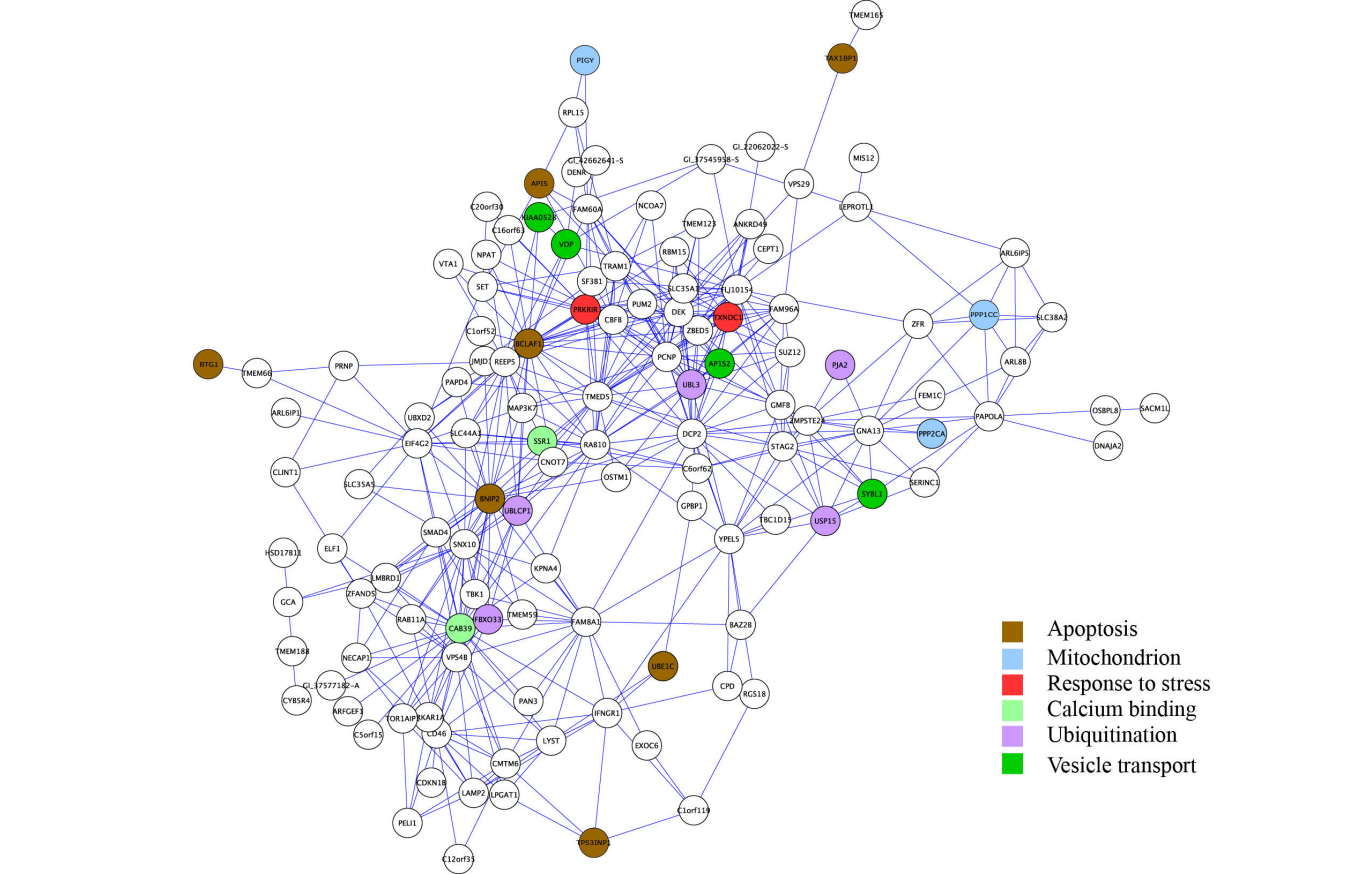
**Network of hub genes in the Blue module colored by gene ontology functional information**. Hub genes are connected by an edge if the correlation between their expression profiles is significant.

Figures 3 and 4 visualizes the connectivity patterns of the 100 intramodular hub genes in the Yellow and Blue modules, respectively. Edges between the intramodular hub genes indicate significant correlations. Both modules contain hub genes involved in apoptosis and protein ubiquitination. Several hub genes in the Blue module are known to be involved in response to stress and vesicle transport. Several hub genes in the Yellow module play an important role in mitochondrial functioning [50].

## Discussion

We use 3 hitherto unpublished blood gene expression data sets to provide the following novel insights regarding ALS. First, both a standard differential expression analysis and WGCNA show that thousands of genes are differentially expressed between patients and controls in peripheral blood even though ALS is a disease primarily affecting neuronal tissue.

Previous studies have shown similar findings in other neurological diseases. In Parkinson's Disease (PD) for example, an assay of eight molecular markers in peripheral whole blood results in higher risk scores for PD patients compared to neurodegenerative disease controls and healthy controls [51]. In Huntington's Disease, a monogenetic disease, expression profile clustering in peripheral blood cells could distinguish not only patients from healthy controls, but also pre-symptomatic from symptomatic patients [25]. In neuronal disease, coordinated repression and expression of large chromosomal regions was found in caudate nucleus and whole blood [52]. In Alzheimer's Disease, changes were detected in the transcriptome of blood mononuclear cells of patients compared to normal controls [26]. These studies and our results support the hypothesis that complex neurological diseases may leave gene expression footprints outside their symptom-related tissue. Since peripheral blood is easily accessible, identification of disease-specific gene expression profiles is a promising step toward the development of biomarkers that can be used for diagnostic and therapeutic purposes. However, our study also shows that more work is needed before blood gene expression profiling will have diagnostic value in clinical practice. We find that gene expression based predictors of ALS (versus healthy controls) have moderate predictive accuracy (80%). Similarly, we find that individual genes have moderate sensitivity, specificity and area under the ROC curve for distinguishing ALS versus healthy controls. ALS biomarker development is especially important for distinguishing ALS patients from those patients mimicking ALS symptoms (e.g. multifocal motor neuropathy, Kennedy's disease and inclusion body myositis) in early stages of disease. For this reason, future biomarker development needs to include larger sample sizes as well as patients with ALS mimic disorders.

Our network analysis reveals that two large co-expression modules (denoted Blue and Yellow) are significantly associated with ALS disease status. In three different and independent sample series we consistently observed that genes within the Blue module were predominantly up regulated in ALS patients, whereas genes in the Yellow module were predominantly down regulated in all three datasets. We did not observe a significant association between modules and other clinical characteristics of ALS patients such as gender, age of onset, site of onset or disease progression. Our analysis does not address whether the two disease-associated modules are causally involved in ALS susceptibility or reflect reactive molecular mechanisms in response to onset of disease. We used Ingenuity pathway analysis to study the functional enrichment of the disease related modules and ALS related genes. We find significant enrichment with regard to functional categories related to genetic disorders including psoriatic arthritis (p = 8.3 × 10^-7^), congenital disorders of glycosylation (p = 2.0 × 10^-4^), Leigh syndrome (2.2 × 10^-4^), neurodegeneration of the nervous system (p = 2.0 × 10^-5^), inflammatory disease (p = 8.3 × 10^-7^) and connective tissue disorders (p = 8.31 × 10^-7^). There are some reports that skin symptoms such as bedsores and a loss of normal elasticity do occur in ALS patients [53] but this has not been observed in patients that are included in this study. Alternatively, it is possible that that these functional categories represent a broader system of inflammation which is thought to be involved in neurodegeneration in general. We compared our findings with previously published gene expression studies of ALS patients. Since these studies were much smaller in size and often based on post mortem neuronal tissue, a direct comparison using network analysis is hardly possible. We therefore compare gene expression patterns at the level of individual genes from ALS associated modules. We noticed a diverse functionality of overlapping genes, and only two genes belonged to a module (E2F3 blue and POLD2 yellow module). The largest overlap between a previous study and our analysis was found with expression patterns in motor cortex of 5 sporadic ALS patients and 3 control subjects [19] with 16 genes coinciding, all down-regulated: ABHD5, PIGB, INTS6, E2F3, BCL6, MFSD1, SAT1, AGTPBP1, MARCH7, TXN, MTMR6, OSBPL11, HSPBAP1, TRIB1, CHUK, CCPG1. Down-regulation of ATP6V1A was found in a second study on motor cortex with 11 sALS patients and 9 control subjects [20]. Overlap with expression data generated from laser captured motor neuron of the spinal cord consisted of 4 genes, all up-regulated (ABL1, USP11, RELA, SPN) [15]. Homogenate of whole Spinal Cord of both sporadic and familial ALS showed differential expression of CD74, CYBA, GYS1, POLD2, ENG, SF1 (upregulated) and CAMLG, SRPK1 (downregulated) [13] and upregulation of VIL2 in a second study [23].

To complement the individual gene information data and to make it a useful resource for others to determine how correlated their gene of interest is to ALS status and disease related modules, we provide a module based gene annotation table (Additional File 4). This table represents for each gene its correlation to the ALS related modules and how associated it is with ALS status.

Network analysis highlights individual intramodular hub genes that are centrally located in the disease related modules. For example, in the Yellow module, Glutathione Synthetase (*GSS*) is worthy of note, since earlier studies have found a decrease of glutathione reductase in erythrocytes of sporadic ALS patients [54]. Glutathione is an important anti-oxidant, mediating defense against lipid peroxidation, shown to be increased in ALS [55]. Other stress responsive genes in the Yellow module are *STIP1 *(*Hsp70/Hsp90*-organizing protein) and *HSP90AB1*, functional in the cytoplasm, and *IDH3B *and *ACO2*, functional in the mitochondrion. A decreased ability of motor neurons to mount a defensive response through up regulation of heat-shock proteins, has been suggested to be part of ALS pathogenesis [56]. Decreased levels of Aconitase-2 (*ACO2*) was found a potential sensitive and early biomarker for mitochondrial oxidant stress [57]. Several apoptotic associated genes were found in the Blue and Yellow modules. *ARHGDIA *inactivates Rho proteins by preventing dissociation of *GDP*. One of the genes that serve as a Guanine Nucleotide exchange factor for *RAB5 *is *ALSIN*. *ALSIN *mutations have been reported in a familial ALS with juvenile onset [58]. *DAXX*, central in the Yellow module, is a modulator of apoptosis of motor neurons in G93A mutant *SOD1 *transgenic mice [59], and is a mediator of the heat-shock protein response [60]. In the Blue network BNIP2 (BCL-2 interacting protein), is a central apoptotic gene. BCL-2 is shown to be involved in the SOD1 mediated cascade leading to motor neuron death [61].

Two genes in the Yellow module are part of the inositol 1,4,5-triphosphate (IP3) pathway, ITPK and PIP5K1A. IP3 regulates the calcium homeostasis in the cell and a polymorphism within a receptor for IP3, ITPR2, is associated with ALS in a sample from the same population from The Netherlands [4]. Increased intracellular calcium levels are shown to be crucial for the induction of motor neuron death [62].

An important question is how well our findings fit with current hypotheses of ALS disease pathophysiology. The most commonly found mutations linked with familial ALS are those in SOD1. The exact (toxic) pathway targeted by mutant SOD1 is not known but the primary function of SOD1 suggests that oxidative stress is involved which could lead to dysfunction of mitochondria and ultimately apoptosis of the neuron. Interestingly, the ALS associated blue and yellow modules are enriched with a number of genes with oxidative and mitochondrial function, suggesting that similar pathways may be involved in familial and sporadic ALS. Recently, mutations in TARDBP and FUS are found in familial ALS [63-65]. These genes both have RNA processing functionality and this category too was significantly enriched in the blue module. Whether dysfunction of RNA processing proteins are part of the same pathogenic pathway as mitochondrial dysfunction is not known, but in our study we found some evidence that both pathways play a role in sporadic ALS.

We compare the findings of WGCNA with those of a standard analysis based on differential expression. As can be seen from our functional enrichment analysis results of different gene lists (Additional File 5), keeping track of module membership leads to statistically more significant enrichment results. While a standard analysis implicates thousands of differentially expressed genes, it fails to recognize that these genes are organized into two large co-expression modules. As a result, a standard analysis indiscriminately averages across modules and dilutes the functional enrichment signal inherent in these modules. In contrast, WGCNA's systems biologic, module-centric approach hones in on disease related pathways and their key drives.

## Conclusion

Weighted gene co-expression network analysis applied to blood gene expression data from ALS patients is combined with Ingenuity Pathway Analysis to implicate disease pathways, molecular mechanisms, and connections to other disorders. Our results suggest that development of an ALS biomarker based on gene expression in peripheral blood may be possible. Moreover, functional insights derived from these genes imply involvement of RNA processing and mitochondrion in sporadic ALS.

For each gene, our module annotation catalogues report the relationship to ALS disease status and its membership to the ALS related co-expression modules in blood. Our results do not point to a single disease pathway. Instead, we find several highly significant pathways and genes in the ALS related modules. Our gene and pathway catalogues are meant to inform additional biological studies.

## Methods

### Sample collection and RNA preparation

Between Jan 1, 2004, and December 31, 2006, all newly diagnosed patients with sporadic ALS at the University Medical Center Utrecht, a referral clinic in The Netherlands, were eligible for recruitment. Diagnosis was made according to the El Escorial Criteria for 'probable' and 'definite' ALS, after exclusion of other conditions [66]. After written informed consent and according to the Institutional Ethics Review Board Protocols, ALS patients and controls were included, frequency matched for age and gender. We obtained a discovery dataset of 30 patients and 30 controls (dataset 1), a replication dataset of same size (dataset 2) and a third dataset with 63 cases and 63 controls (dataset 3) amounting to a total of 123 ALS patients and 123 controls. Onset of disease was defined as the time of initial weakness, dysarthria or dysphagia. The controls are genetically unrelated individuals accompanying the patient during their outpatient visits. Clinical characteristics of patients and controls for the three datasets are shown in Table 1.

Messenger RNA was obtained from peripheral blood samples that were drawn when the patients first visited the motor neuron disease outpatient clinic. Blood from patients and controls was drawn in the morning. For isolation and purification of mRNA from whole blood the PAXgene extraction kit (Qiagen) has been used for all samples. PAXgene tubes contain a proprietary reagent that immediately stabilizes intracellular mRNA, thus reducing mRNA degradation and inhibiting gene induction after phlebotomy. The mRNA isolated with this protocol comes from all blood cells, including polymorphonuclear leukocytes, mononuclear cells, platelets and red blood cells. Total leukcocyte counts and leukocyte differentiation showed no significant differences between patients and controls. RNA was isolated according to the manufacturer's instructions including an optional DNase digestion step. The standardized mRNA isolation procedure guarantees high quality non-degraded mRNA. Total mRNA was quantified using spectrophotometry. Quality of total RNA was checked using Agilent 2100 Bioanalyzer.

### Gene-expression profiling

The Illumina Sentrix HumanRef-8 Expression BeadChip with >22,000 current RefSeq curated gene targets was used to obtain gene expression data. Cubic spline normalization was performed in Illumina's software package Beadstudio. After normalization data was imported into R .

About one third of the genes (8,000 genes) were found to be significantly expressed in peripheral blood at measurable levels (Bead studio mean detection level of p < 0.05). These genes were selected as starting point of the co-expression network analysis described in the following.

### Weighted gene co-expression network analysis (WGCNA)

We constructed weighted gene co-expression networks as previously described [42]. The determination of weighted co-expression starts by calculating a correlation matrix containing all pairwise Pearson correlations between all probe sets across all subjects. We define coexpression networks as undirected, weighted gene networks. The nodes of such a network correspond to gene expressions, and edges between genes are determined by the pairwise Pearson correlations between gene expressions. By raising the absolute value of the Pearson correlation to a power β ≥ 1 (soft thresholding), the weighted gene coexpression network construction emphasizes large correlations at the expense of low correlations. Specifically, *a*_*ij *_= |cor(*x*_*i*_, *x*_*j*_)|^β ^represents the adjacency of an (unsigned) weighted gene co-express network. We used the scale free topology criterion to choose the soft threshold β = 6. A major advantage of weighted networks is that they are highly robust with regard to the choice of the parameter β.

Unlike unweighted networks that use a hard threshold to dichotomize the correlation matrix, the soft thresholding of weighted gene co-expression networks preserves the continuous nature of the gene co-expression information, leading to highly robust results [42] and allowing for a simple geometric interpretation of network concepts [44,67]. To organize genes (probes) into modules, we used the topological overlap measure as a robust measure of interconnectedness in a hierarchical cluster analysis [68-70].

### Connectivity and module membership measures

Here we focus on connectivity (centrality) measures that are useful within the WGCNA context. Whole network connectivity *k*(*i*) is the sum of the connection strengths between a particular gene *x*_*i*_and all other genes in the network , where *N *refers to the set of network genes. However, we have found that intramodular connectivity *k*^*q*^(*i*) is a biologically more meaningful measure in the context of our module-based analysis. It is computed from the sum of the connection strengths between a particular gene and all other genes in the module , where *q *refers to a specific module. Another measure of connectivity is the (fuzzy) module membership measure *MM*^*q*^(*i*), which is sometimes referred to as eigengene-based connectivity kME; for the i-th gene, it is defined as the correlation between the i-th gene expression profile *x*_*i *_and the *q*-th module eigengene", *ME*^*q*^.

*MM*^*q*^(*i*) = Cor(*x*_*i*_, *ME*^*q*^), where larger absolute values indicate greater similarity between a gene *x*_*i *_and the *q*-th module eigengene. One can show that the module eigengene-based connectivity measure is highly correlated with intramodular connectivity [44] but the module membership measure enjoys several advantages including that its definition can be easily extended to genes outside the original module and that it allows one to use a correlation test to assess the statistical significance (p-value) of module membership. WGCNA also outputs the corresponding correlation test p-value for module membership (denoted by PvalueMMblue).

The networkScreening function in the WGCNA R package computes the gene selection score p.weighted based on gene significance and on module membership [45]. Analogous to a p-value, the smaller the value of p.weighted the stronger is the evidence that the gene is a disease related hub gene.

### Definition of Four Gene Lists

For a comparative functional enrichment analysis with Ingenuity, we considered four different gene lists: first, a list of 500 genes with the highest evidence of module membership in the blue module, an analogous list of 500 genes with high module membership in the yellow module, a third list of genes with lowest score p.weighted, and a fourth list of 500 most differentially expressed genes according to a standard analysis.

For the Blue and Yellow modules, we selected gene lists of 500 genes with module membership p-values smaller than 10^-22 ^and corresponding local false discovery rates (q-values) smaller than 10^-5^. Genes on list 1 and 2 were sorted according to the mean module membership in the Blue and Yellow Module, respectively. For example, the 500 Yellow module genes had mean membership values larger than 0.64.

A third list of 500 genes was selected using the WGCNA gene selection score p.weighted described above.

A fourth list of 500 genes was selected based on the average Student T-test statistic (of differential expression) across the 3 independent data sets. Thus, this fourth list of genes represents the results of a standard differential expression analysis.

### Finding ALS-related modules

To incorporate ALS disease status into the co-expression network, we first defined a measure of gene significance (GS). Abstractly speaking, the higher the i-th gene's |GS(i)|, the greater its biological significance. For the i-th gene, we define GS.ALS(i) as the absolute value of the Student T-test statistic for testing differential expression between ALS patients and controls. We defined two related measures of module significance. The first measure of module significance is simply the average gene significance measure across module genes. The second measure of module significance is defined as the eigengene significance, i.e. the correlation between the module eigengene and ALS status. It turns out that the two measures of module membership are highly related [44] but the eigengene significance measure has the advantage of allowing one to use a correlation test for computing a corresponding p-value. Since we found only 5 modules, a Bonferroni correction of these p-values requires that one multiply the corresponding p-values by 5.

### Replication of module definition

To replicate and validate the existence of these modules, we mapped the module color assignment of the discovery dataset into the second and subsequently into the third independent datasets. Hub genes are defined as genes with the highest intramodular connectivity, i.e. genes that are most central within the module. Once modules were identified, the module eigengene (ME; i.e. the first principal component of the expression values across subjects) was calculated using all probe sets in each module. The MEs were then correlated to relevant clinical traits using the Pearson correlation and Cox regression. Our custom-made R software function for weighted gene co-expression network analysis and the data are available upon request.

Networks of disease related hub genes were generated by using the R library *MetaNetwork *to create input files for Cytoscape .

### Finding ALS related genes

Differential expression of genes was also calculated with a multivariate linear regression model including gender, age and batch. To correct for multiple testing, we used a 5% false discovery rate (Benjamini Hochberg). For each gene sensitivity, specificity and area under the receiving operating curve (AUC) was calculated. The first two measurements were calculated using the mean expression as a cut-off. Combining datasets 1, 2 and 3 was performed using the R package *Lumi*. Raw data without background correction were normalized with the *expresso *function.

### Functional Enrichment Analysis

We used the Ingenuity Pathways Analysis (IPA, ) software to determine whether a set of genes (e.g. top 500 module genes) were significantly enriched with known gene ontologies. The software computes a Fisher exact test p-value. The IPA p-values are not corrected for multiple testing.

We also used Gene Ontology  and Webgestalt to investigate enrichment of specific functions within modules of co-expressed genes that were associated with disease status.

## List of Abbreviations

ALS: Amyotrophic Lateral Sclerosis; ME: module eigengene; MM: module membership measure; SOD1: superoxide dismutase-1; WGCNA: weighted gene co-expression network analysis.

## Competing interests

The authors declare that they have no competing interests.

## Authors' contributions

CGJS, JV, JHJW, LHB, and RAO designed research; CGJS, PWJV, MAE, HMB and JDY, performed research; CGJS, SH, TFF, PL and JV analyzed the data; CGJS, JV, LHB., SH. and RAO wrote the paper. All authors read and approved the final manuscript.

## Supplementary Material

Additional file 1 **Differentially expressed genes between ALS cases and controls**. Standard differential expression analysis that reports genes that are differentially expressed between ALS and healthy controls in the discovery set. At a false discovery rate cut-off of 0.05, 2300 probes were differentially expressed between ALS cases and controls. Click here for file

Additional file 2 **Reproducibility of co-expression modules across three data sets**. Robustness of module detection across three networks denoted by R1, R2, and R3 corresponding to data sets 1, 2, and 3, respectively. Genes are colored according to their module assignment in the discovery set (R1) where five distinct branches (modules) were found (colored in Blue, Green, Red, Turquoise and Yellow). The fact that most genes of the same color tend to cluster together in data sets R2 and R3 reflects that these modules can also be found in these test data sets. The ALS related modules (Blue and Yellow) are preserved across all three data sets but the Red and Green module can only be found in data sets R1 and R2. An alternatively way of studying module preservation is afforded by module membership measures, see Additional File 3. Click here for file

Additional file 3 **Scatterplots of module membership measures between the three data sets**. Genes are colored by their module assignment in the discovery set. The axes correspond to module membership measures in the different data sets. MMBlueR1, MMBlueR2, MMBlueR3 denotes the module membership with regard to the Blue module in data sets 1, 2, and 3, respectively. Note that genes with high positive (or high negative) Blue module membership in data set 1 tend to have a similar value in data sets 2 and 3. The same applies for module membership with regard to the Yellow module. Also note that the Blue genes tend to have negative module membership values with regard to the yellow module and vice versa. This reflects the fact that the Blue and Yellow module eigengenes are anti-correlated. The fact that the Blue and Yellow module membership values are preserved across the three data sets reflects the fact that these modules can be detected in all three data sets (Additional File 2). Click here for file

Additional file 4 **Module membership annotation table**. The table provides the module membership annotation with regard to different modules in each of the 3 independent data sets. Values are reported for the 15463 probes for which there was significant evidence of presence in blood. MMBlueR1 denotes the module membership value to the Blue module in data set 1, i.e. the correlation between expression profiles and the Blue module eigengene. PvalueMMblueR1 denotes the corresponding correlation test p-value. The mean module membership with regard to the Blue module is referred to as MeanMMblue. Click here for file

Additional file 5 **Comprehensive functional enrichment results of an Ingenuity Pathways Analysis**. The figure shows the functional enrichment results of an Ingenuity Pathways Analysis for four different gene lists comprised of 500 genes each. Specifically, functional enrichment is reported for 500 genes with highest membership to the Blue Module (blue horizontal bars), highest membership to the Yellow Module (yellow bars), lowest WGCNA gene selection score p.weighted (turquoise bars), and most significant Student T-test (black bars). Click here for file

Additional file 6 **Selected functional enrichment results of an Ingenuity Pathways Analysis**. This figure represents a selected view of Additional File 5. Ingenuity Pathways Analysis shows selected overrepresented categories in the 3 network related lists comprised of 500 genes each. Specifically, functional enrichment is reported for 500 genes with highest membership to the Blue Module, the Yellow Module, and most significant WGCNA gene selection score (p.weighted). Click here for file

Additional file 7 **Results of an Ingenuity biomarker analysis**. This Excel file reports the results of an Ingenuity biomarker analysis where we analyzed the top 500 genes with most significant WGCNA score (p.weighted). The table reports in which tissues matching genes are known to be expressed and known drug targets for matching genes. Click here for file

Additional file 8 **Intramodular hub genes in the ALS related co-expression modules**. The table reports hub genes in the Blue and Yellow module with possible function in pathogenesis in ALS based on Gene Ontology function. Selection was made within the top 100 most highly connected genes in both modules and genes were categorized by their Gene Ontology (GO) function. Module membership is listed as well as fold change, Area Under the ROC Curve (AUC), sensitivity (Sensit), Specificity (Specif) and overall p-value of differential expression. Click here for file
