# Supplementary figures and images for: Weighted gene co-expression network analysis of the peripheral blood from Amyotrophic Lateral Sclerosis patients
Source: BMC Genomics. 2009 Aug 27;10:405. doi: 10.1186/1471-2164-10-405 (PMC2743717; doi:10.1186/1471-2164-10-405)

ALS R1 Network

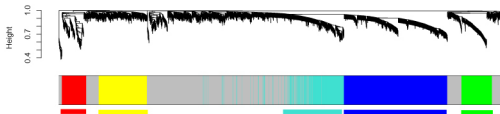

ALS R2 Network

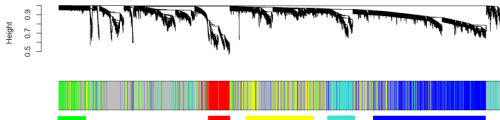

ALS R3 Network

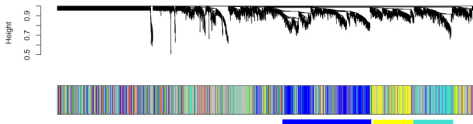

Supplement: Additional file 2 — Reproducibility of co-expression modules across three data sets. Robustness of module detection across three networks denoted by R1, R2, and R3 corresponding to data sets 1, 2, and 3, respectively. Genes are colored according to their module assignment in the discovery set (R1) where five distinct branches (modules) were found (colored in Blue, Green, Red, Turquoise and Yellow). The fact that most genes of the same color tend to cluster together in data sets R2 and R3 reflects that these modules can also be found in these test data sets. The ALS related modules (Blue and Yellow) are preserved across all three data sets but the Red and Green module can only be found in data sets R1 and R2. An alternatively way of studying module preservation is afforded by module membership measures, see Additional File 3. [file 1471-2164-10-405-S2.pdf]

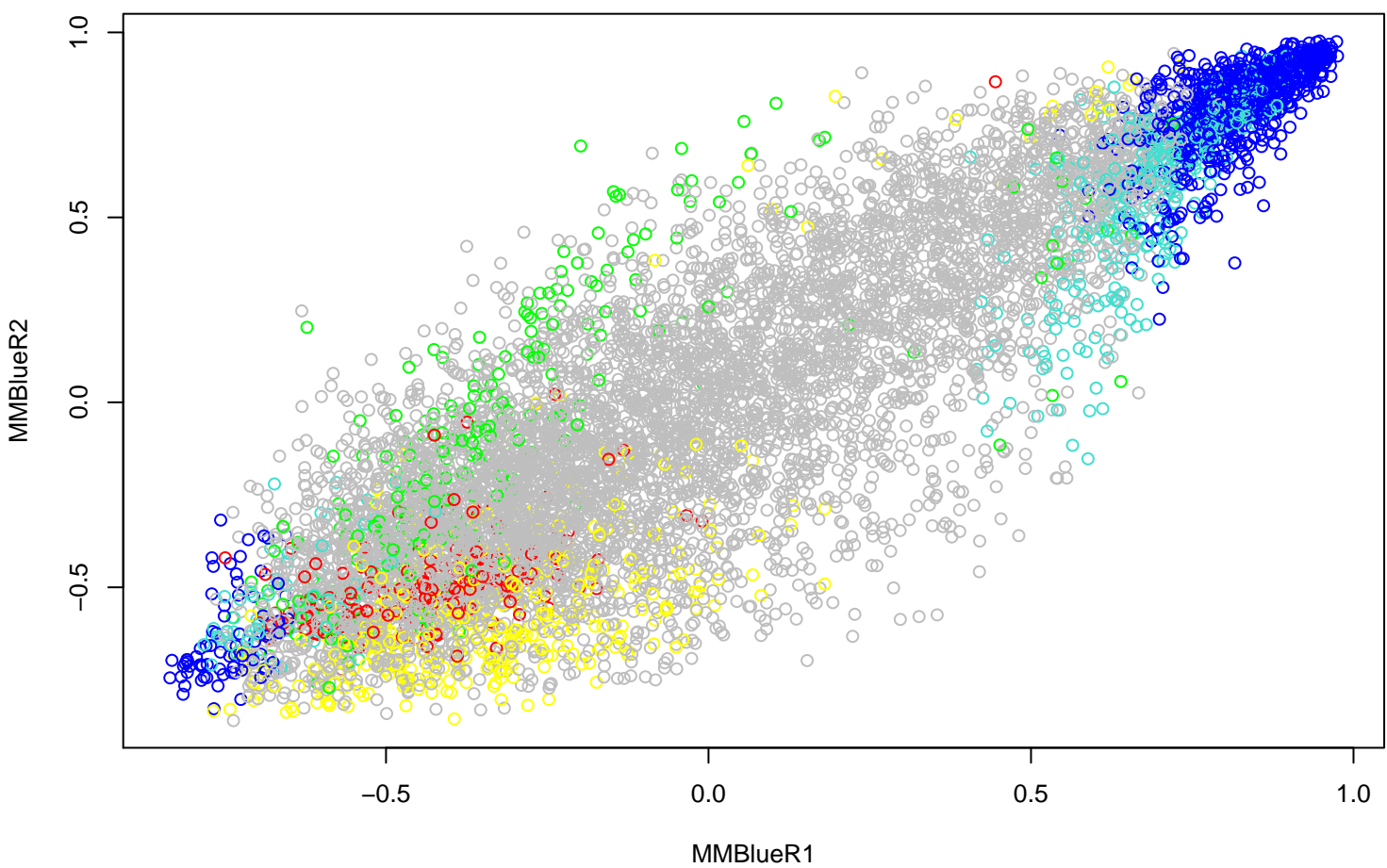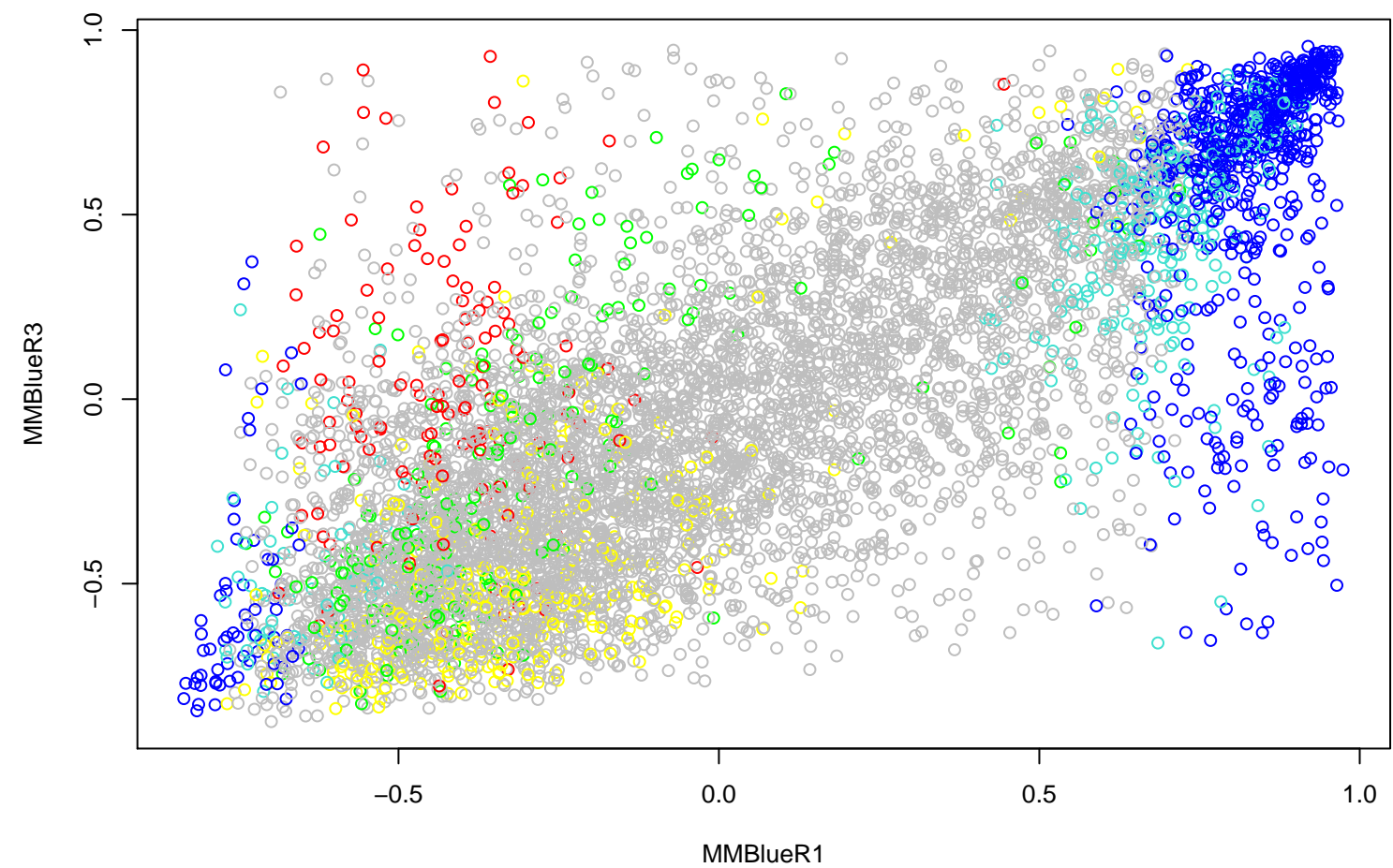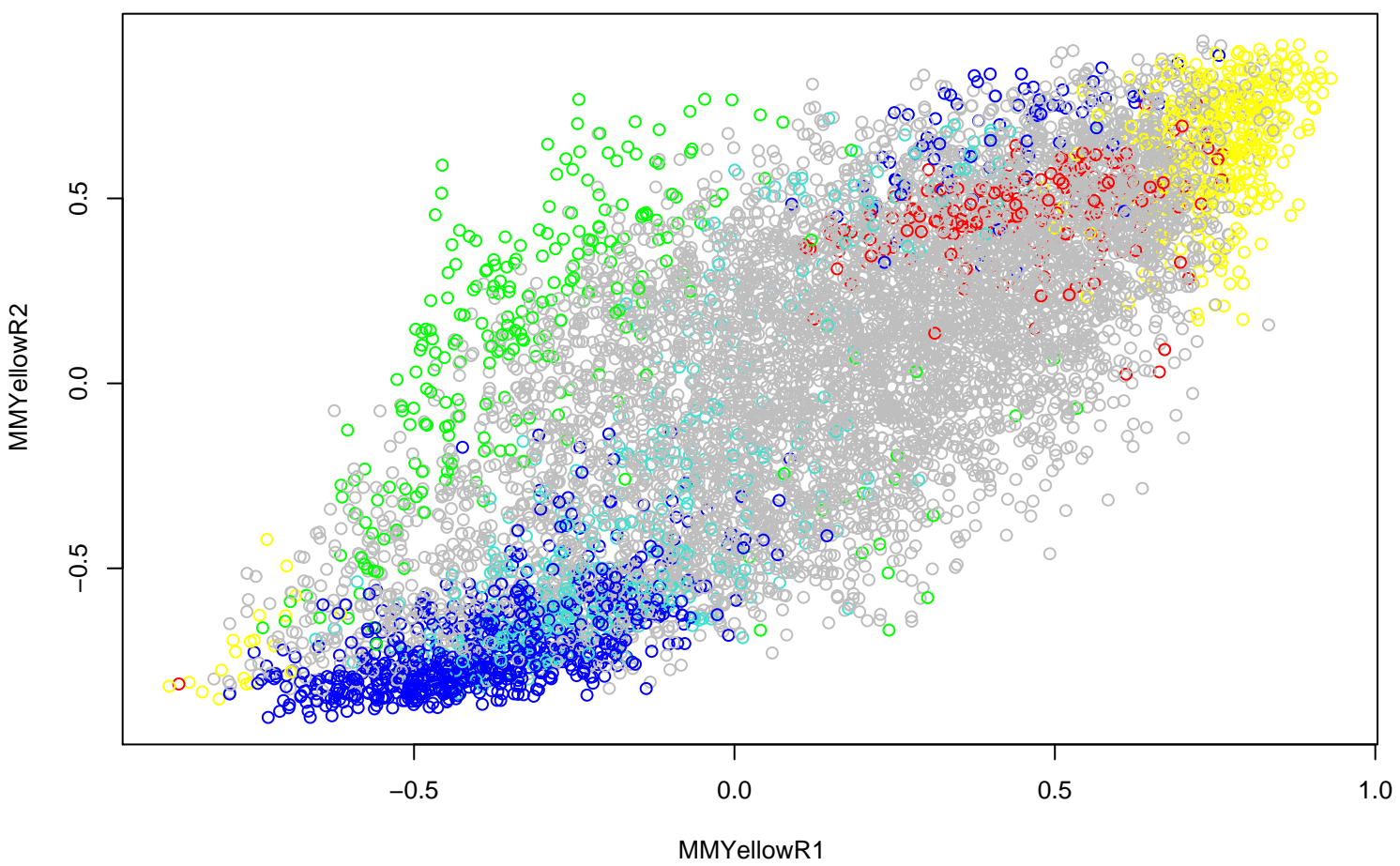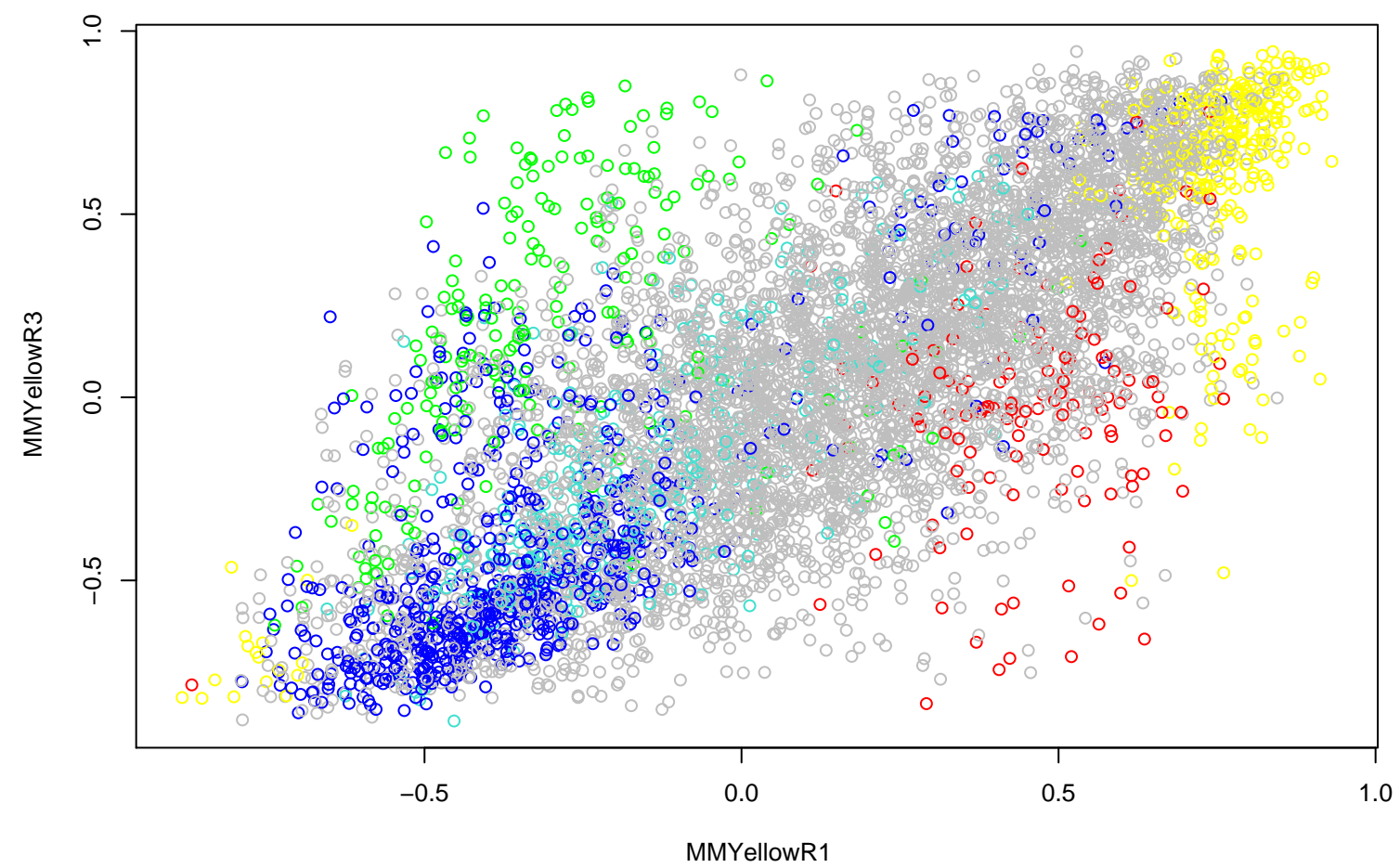

Supplement: Additional file 3 — Scatterplots of module membership measures between the three data sets. Genes are colored by their module assignment in the discovery set. The axes correspond to module membership measures in the different data sets. MMBlueR1, MMBlueR2, MMBlueR3 denotes the module membership with regard to the Blue module in data sets 1, 2, and 3, respectively. Note that genes with high positive (or high negative) Blue module membership in data set 1 tend to have a similar value in data sets 2 and 3. The same applies for module membership with regard to the Yellow module. Also note that the Blue genes tend to have negative module membership values with regard to the yellow module and vice versa. This reflects the fact that the Blue and Yellow module eigengenes are anti-correlated. The fact that the Blue and Yellow module membership values are preserved across the three data sets reflects the fact that these modules can be detected in all three data sets (Additional File 2). [file 1471-2164-10-405-S3.pdf]

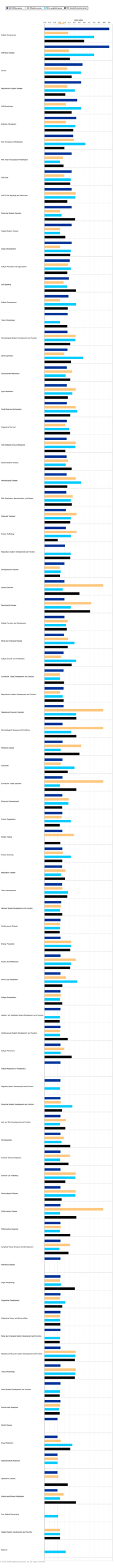

Supplement: Additional file 5 — Comprehensive functional enrichment results of an Ingenuity Pathways Analysis. The figure shows the functional enrichment results of an Ingenuity Pathways Analysis for four different gene lists comprised of 500 genes each. Specifically, functional enrichment is reported for 500 genes with highest membership to the Blue Module (blue horizontal bars), highest membership to the Yellow Module (yellow bars), lowest WGCNA gene selection score p.weighted (turquoise bars), and most significant Student T-test (black bars). [file 1471-2164-10-405-S5.pdf]

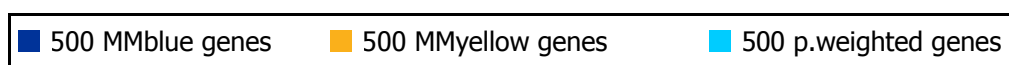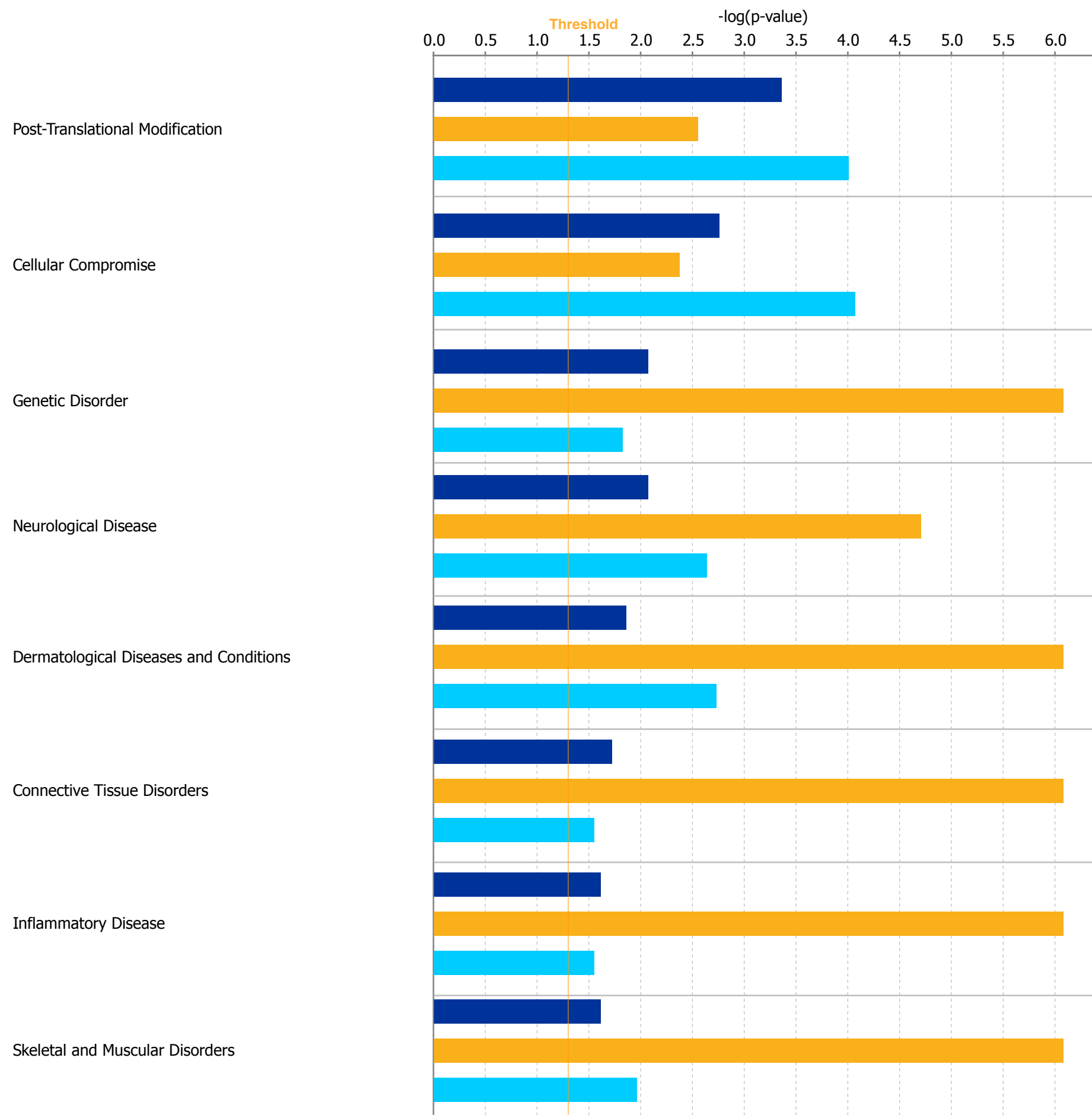

Supplement: Additional file 6 — Selected functional enrichment results of an Ingenuity Pathways Analysis. This figure represents a selected view of Additional File 5. Ingenuity Pathways Analysis shows selected overrepresented categories in the 3 network related lists comprised of 500 genes each. Specifically, functional enrichment is reported for 500 genes with highest membership to the Blue Module, the Yellow Module, and most significant WGCNA gene selection score (p.weighted). [file 1471-2164-10-405-S6.pdf]
