# Supplementary material for: Weighted gene co-expression network analysis of the peripheral blood from Amyotrophic Lateral Sclerosis patients
Source: BMC Genomics. 2009 Aug 27;10:405. doi: 10.1186/1471-2164-10-405 (PMC2743717; doi:10.1186/1471-2164-10-405)
Supplement: Additional file 8 — Intramodular hub genes in the ALS related co-expression modules. The table reports hub genes in the Blue and Yellow module with possible function in pathogenesis in ALS based on Gene Ontology function. Selection was made within the top 100 most highly connected genes in both modules and genes were categorized by their Gene Ontology (GO) function. Module membership is listed as well as fold change, Area Under the ROC Curve (AUC), sensitivity (Sensit), Specificity (Specif) and overall p-value of differential expression. [file 1471-2164-10-405-S8.doc]

**Additional File 8. Hub genes in the Blue and Yellow module with possible function in pathogenesis in ALS based on Gene Ontology function.** Selection was made within the top 100 most highly connected genes in both modules and genes were categorized by their Gene Ontology (GO) function. Module membership is listed as well as fold change, Area Under the ROC Curve (AUC), sensitivity (Sensit), Specificity (Specif) and overall p-value of differential expression.

| **GO – category** | **Probe ID** | **Symbol** | **Definition** | **Fold**  **change** | **AUC** | **Sensit** | **Specif** | **P value** | **Module** |
| --- | --- | --- | --- | --- | --- | --- | --- | --- | --- |
| **Apoptosis** | GI_38788404-S | HD | huntingtin (Huntington disease) | -1.13 | 0.69 | 0.58 | 0.58 | 1.02e-16 | yellow |
|  | GI_31542491-S | DAXX | death-associated protein 6 | -1.20 | 0.79 | 0.72 | 0.72 | 5.42e-16 | yellow |
|  | GI_34147601-S | ARHGDIA | Rho GDP dissociation inhibitor (GDI) alpha | -1.23 | 0.71 | 0.63 | 0.64 | 3.03e-15 | yellow |
|  | GI_4757855-S | BNIP2 | Homo sapiens BCL2/adenovirus E1B 19kDa interacting protein 2 | 1.19 | 0.72 | 0.68 | 0.65 | 4.29e-12 | blue |
|  | GI_7661957-S | BCLAF1 | Homo sapiens BCL2-associated transcription factor 1 | 1.25 | 0.74 | 0.70 | 0.67 | 5.40e-11 | blue |
|  | GI_38045945-A | UBE1C | Homo sapiens ubiquitin-activating enzyme E1C | 1.17 | 0.72 | 0.67 | 0.66 | 8.44e-10 | blue |
|  | GI_38202242-S | YARS | tyrosyl-tRNA synthetase | -1.13 | 0.63 | 0.53 | 0.52 | 7.95e-09 | yellow |
|  |  |  |  |  |  |  |  |  |  |
| **Mitochondrion** | GI_28178815-A | IDH3B | isocitrate dehydrogenase 3 (NAD+) beta (IDH3B), nuclear gene encoding mitochondrial protein | -1.16 | 0.67 | 0.52 | 0.52 | 1.63e-14 | yellow |
|  | GI_4506016-S | PPP2CA | Homo sapiens protein phosphatase 2 (formerly 2A), catalytic subunit, alpha isoform | 1.25 | 0.75 | 0.67 | 0.68 | 2.94e-14 | blue |
|  | GI_20149593-S | HSP90AB1 | heat shock protein 90kDa alpha (cytosolic), class B member 1 | -1.18 | 0.72 | 0.65 | 0.66 | 2.28E-13 | yellow |
|  | GI_28416952-S | ACSS1 | acyl-CoA synthetase short-chain family  member 1 | -1.15 | 0.66 | 0.56 | 0.55 | 8.88e-13 | yellow |
|  | GI_7661547-S | C2orf25 | chromosome 2 open reading frame 25 | 1.16 | 0.70 | 0.67 | 0.66 | 1.10e-12 | yellow |
|  | GI_4501866-S | ACO2 | aconitase 2, mitochondrial | -1.12 | 0.65 | 0.54 | 0.54 | 2.07e-10 | yellow |
|  | GI_29550827-S | ACBD3 | Homo sapiens acyl-Coenzyme A binding domain containing 3 | 1.11 | 0.69 | 0.66 | 0.66 | 4.05e-09 | blue |
|  | GI_4506006-S | PPP1CC | Homo sapiens protein phosphatase 1, catalytic subunit, gamma isoform | 1.26 | 0.71 | 0.69 | 0.67 | 1.37E-08 | blue |
|  | GI_34147449-S | PIGY | Homo sapiens phosphatidylinositol glycan, class Y | 1.15 | 0.69 | 0.64 | 0.62 | 2.22E-07 | blue |
|  | GI_7662347-S | MLXIP | Homo sapiens MLX interacting protein | -1.08 | 0.58 | 0.50 | 0.50 | 2.69E-03 | yellow |
|  | GI_40255138-S | D2HGDH | Homo sapiens hypothetical protein | -1.08 | 0.57 | 0.51 | 0.50 | 1.73E-02 | yellow |
|  |  |  |  |  |  |  |  |  |  |
| **Response to stress** | GI_5803180-S | STIP1 | stress-induced-phosphoprotein 1 | -1.22 | 0.72 | 0.63 | 0.61 | 5.93e-16 | yellow |
|  | GI_19923267-S | PRKRIR | Homo sapiens protein-kinase, interferon-inducible double stranded RNA dependent inhibitor | 1.20 | 0.74 | 0.70 | 0.66 | 9.18e-12 | blue |
|  | GI_19923408-S | SERP1 | Homo sapiens stress-associated endoplasmic reticulum protein 1 | 1.11 | 0.71 | 0.69 | 0.63 | 2.27e-11 | blue |
|  | GI_30581166-S | GSS | glutathione synthetase | -1.10 | 0.66 | 0.50 | 0.50 | 1.95e-08 | yellow |
|  | GI_34222132-S | TXNDC | Homo sapiens thioredoxin domain containing | 1.20 | 0.69 | 0.69 | 0.64 | 8.69e-08 | blue |
|  |  |  |  |  |  |  |  |  |  |
| **Inositol trisphosphate** | GI_38569399-S | ITPKB | inositol 1,4,5-trisphosphate 3-kinase B | -1.18 | 0.74 | 0.69 | 0.67 | 3.46e-17 | yellow |
|  | GI_31317308-S | PIP5K1C | phosphatidylinositol-4-phosphate 5-kinase, type I, gamma | -1.16 | 0.79 | 0.74 | 0.71 | 4.16e-16 | yellow |
|  |  |  |  |  |  |  |  |  |  |
| **Vesicle transport** | GI_27545446-S | SYBL1 | Homo sapiens synaptobrevin-like 1 | 1.16 | 0.74 | 0.71 | 0.68 | 9.16e-12 | blue |
|  | GI_4505540-S | VDP | Homo sapiens vesicle docking protein p115 | 1.12 | 0.67 | 0.61 | 0.60 | 2.41e-08 | blue |
|  | GI_22027654-S | AP1S2 | Homo sapiens adaptor-related protein complex 1, sigma 2 subunit (AP1S2), mRNA. | 1.32 | 0.69 | 0.66 | 0.65 | 8.79e-08 | blue |
|  | GI_29789059-S | KIAA0528 | Homo sapiens KIAA0528 | 1.15 | 0.70 | 0.67 | 0.63 | 7.53e-08 | blue |
|  |  |  |  |  |  |  |  |  |  |
| **Ubiquitination** | GI_24307990-S | PARC | Homo sapiens p53-associated parkin-like cytoplasmic protein | -1.12 | 0.83 | 0.73 | 0.74 | 1.38e-20 | yellow |
|  | GI_14971416-S | TRIM28 | Homo sapiens tripartite motif-containing 28 | -1.17 | 0.79 | 0.76 | 0.74 | 1.18e-16 | yellow |
|  | GI_41281511-S | PJA2 | Homo sapiens praja 2, RING-H2 motif containing | 1.48 | 0.77 | 0.73 | 0.71 | 7.10e-14 | blue |
|  | GI_40805103-S | TOPORS | Homo sapiens topoisomerase I binding, arginine/serine-rich | 1.10 | 0.73 | 0.68 | 0.64 | 2.27e-13 | yellow |
|  | GI_42558257-S | FBXO33 | Homo sapiens F-box protein 33 | 1.18 | 0.75 | 0.68 | 0.66 | 7.31e-13 | blue |
|  | GI_21450801-S | UBLCP1 | Homo sapiens ubiquitin-like domain containing CTD phosphatase 1 | 1.18 | 0.77 | 0.70 | 0.68 | 1.12e-12 | blue |
|  | GI_14149626-S | USP15 | Homo sapiens ubiquitin specific peptidase 15 | 1.17 | 0.76 | 0.74 | 0.69 | 1.35e-12 | blue |
|  | GI_21265128-A | USP21 | ubiquitin specific peptidase 21 | -1.07 | 0.73 | 0.68 | 0.69 | 3.58e-12 | yellow |
|  | GI_40254846-S | UBL3 | Homo sapiens ubiquitin-like 3 | 1.16 | 0.70 | 0.65 | 0.62 | 5.90e-09 | blue |
|  | GI_16445440-S | TRIM26 | Homo sapiens tripartite motif-containing 26 | -1.13 | 0.62 | 0.52 | 0.51 | 2.42e-07 | yellow |
|  | GI_21362004-S | FBXO31 | Homo sapiens F-box protein 31 | -1.10 | 0.64 | 0.52 | 0.52 | 3.26e-07 | yellow |
|  |  |  |  |  |  |  |  |  |  |
| **Calcium binding** | GI_4502564-S | CAPNS1 | calpain, small subunit 1 | -1.24 | 0.77 | 0.72 | 0.69 | 3.24e-15 | yellow |
|  | GI_34147653-S | SLC3A2 | Homo sapiens solute carrier family 3 | -1.12 | 0.69 | 0.62 | 0.62 | 2.22e-11 | yellow |
|  | GI_6552340-S | SSR1 | Homo sapiens signal sequence receptor, alpha | 1.22 | 0.74 | 0.65 | 0.66 | 4.17e-11 | blue |
|  | GI_19745179-S | CAB39 | Homo sapiens calcium binding protein 39 | 1.33 | 0.70 | 0.65 | 0.65 | 3.25e-09 | blue |
|  | GI_7662035-S | SPOCK2 | Homo sapiens sparc/osteonectin, cwcv and kazal-like domains proteoglycan | -1.25 | 0.62 | 0.59 | 0.58 | 3.86e-09 | yellow |
|  | GI_4507190-S | SPTAN1 | Homo sapiens spectrin, alpha, non-erythrocytic 1 | -1.27 | 0.66 | 0.52 | 0.51 | 1.89e-08 | yellow |
|  | GI_41281560-S | CLSTN1 | Homo sapiens calsyntenin 1 | -1.07 | 0.59 | 0.51 | 0.50 | 1.78e-02 | yellow |
|  |  |  |  |  |  |  |  |  |  |
| **Other** | GI_31795542-S | GMFB | Homo sapiens glia maturation factor, beta | 1.16 | 0.72 | 0.73 | 0.67 | 6.06e-11 | blue |
|  | GI_7669496-S | ARL6IP5 | Homo sapiens ADP-ribosylation-like factor 6 interacting protein 5 | 1.33 | 0.71 | 0.71 | 0.66 | 5.85e-09 | blue |
|  | GI_6715588-S | ARFGEF1 | Homo sapiens ADP-ribosylation factor guanine nucleotide-exchange factor 1 | 1.11 | 0.70 | 0.64 | 0.64 | 9.64e-10 | blue |
|  | GI_26080432-S | DPP9 | dipeptidyl-peptidase 9 | -1.20 | 0.69 | 0.58 | 0.58 | 4.05e-14 | yellow |
|  | GI_33350931-S | DYNC1H1 | dynein, cytoplasmic 1, heavy chain 1 | -1.21 | 0.79 | 0.73 | 0.71 | 5.86e-17 | yellow |
|  | GI_32699044-S | PDDC1 | Parkinson disease 7 domain containing 1 | -1.13 | 0.65 | 0.54 | 0.54 | 7.14e-12 | yellow |
